# Physical-Mechanical Behavior and Water-Barrier Properties of Biopolymers-Clay Nanocomposites

**DOI:** 10.3390/molecules26216734

**Published:** 2021-11-07

**Authors:** Heidy Lorena Calambas, Abril Fonseca, Dayana Adames, Yaneli Aguirre-Loredo, Carolina Caicedo

**Affiliations:** 1 Grupo de Investigación en Desarrollo de Materiales y Productos, Centro Nacional de Asistencia Técnica a la Industria (ASTIN), SENA, Cali 760003, Colombia; hlcalambas@misena.edu.co; 2 Centro de Investigación en Química Aplicada (CIQA), Blvd. Enrique Reyna Hermosillo 140, Saltillo 25294, Coahuila, Mexico; abril.fonseca@ciqa.edu.mx; 3 CONACYT-CIQA, Blvd. Enrique Reyna Hermosillo 140, Saltillo 25294, Coahuila, Mexico; 4 Semillero de Investigación en Química Aplicada (SEQUIA), Facultad de Ciencias Básicas, Universidad Santiago de Cali, Pampa linda, Santiago de Cali 760035, Colombia; yicel.adames00@usc.edu.co; 5 Grupo de Investigación en Química y Biotecnología (QUIBIO), Facultad de Ciencias Básicas, Universidad Santiago de Cali, Pampalinda, Santiago de Cali 760035, Colombia

**Keywords:** biodegradable polymers, nanocomposite, ultrasound-assisted, mechanical properties, montmorillonite

## Abstract

The preparation and characterization of biodegradable films based on starch-PVA-nanoclay by solvent casting are reported in this study. The films were prepared with a relation of 3:2 of starch:PVA and nanoclay (0.5, 1.0, and 1.5% *w*/*v*), and glycerol as plasticizer. The nanoclays before being incorporated in the filmogenic solution of starch-PVA were dispersed in two ways: by magnetic stirring and by sonication. The SEM results suggest that the sonication of nanoclay is necessary to reach a good dispersion along the polymeric matrix. FTIR results of films with 1.0 and 1.5% *w*/*v* of sonicated nanoclay suggest a strong interaction of hydrogen bond with the polymeric matrix of starch-PVA. However, the properties of WVP, tensile strength, percentage of elongation at break, and Young’s modulus improved to the film with sonicated nanoclay at 0.5% *w*/*v*, while in films with 1.0 and 1.5% *w*/*w* these properties were even worse than in film without nanoclay. Nanoclay concentrations higher than 1.0 *w*/*v* saturate the polymer matrix, affecting the physicochemical properties. Accordingly, the successful incorporation of nanoclays at 0.5% *w*/*v* into the matrix starch-PVA suggests that this film is a good candidate for use as biodegradable packaging.

## 1. Introduction

In recent years, there has been a worldwide increase in societal interest in using environmentally friendly plastic materials, which has resulted in an increase in the number of polymers of organic origin being researched and developed. These new materials are intended to be a viable alternative to petroleum-derived plastics, which have a long degradation or disintegration time as their main drawback [1,2]. Starch is one of the most promising natural polymers for developing biodegradable packaging materials, owing to its abundance in nature, low cost, and short periods of degradability [3].

Although starch is abundant in nature, this polymer presents some challenges or disadvantages that must be overcome if it is to be used as a raw material to obtain new sustainable packaging. Pure starch-based materials have been shown to have poor mechanical properties, high water solubility, and poor gas barrier properties, making them unsuitable for use as packaging. However, when certain additives, such as a plasticizer are added to the starch, the material becomes thermoplastic, which improves the material’s processing and functional behavior [4]. A plasticizer increases the flexibility of starch-based materials due to reducing the interaction of polymer-polymer hydrogen bonds, improving interfacial adhesion, and decreasing intermolecular binding sites starch in granules’ crystalline regions [5].

Even though the scientific and technological advances that have been developed to improve the physical-mechanical properties of thermoplastic starch (TPS), the materials continue to present some challenges that limit their applicability due to their high susceptibility to humidity and the retrogradation process [6]. To address these shortcomings, various strategies have been implemented, the most notable of which is the combination of starch with other less hydrophilic polymers to produce materials with improved properties. In comparison to pure starch materials, some TPS/polyvinyl alcohol (PVA) mixtures have been evaluated, which have generated materials with a gas barrier behavior and improved mechanical performance, as well as a significant reduction in their water absorption capacity. In the study carried out by Gómez-Aldapa et al., biodegradable materials were made of potato starch and PVA; the authors reported a significant improvement in the films’ water vapor barrier and mechanical performance [7]. In addition to providing an improvement in the physicomechanical properties of starch-based films, the incorporation of PVA has been shown to generate important stability to the polymeric matrix, which inhibits the physical changes caused by the aging of the material, which is a critical parameter to consider in this type of materials [8]. Therefore, it is observed that the manufacture of packaging materials based on starch-PVA mixtures can be a viable option for use in the packaging of products in general, among which the food industry stands out due to its high demand.

The improvement of starch-based packaging materials has been sought with the help of nanotechnology, in addition to resorting to the development of mixtures with other polymers. In this sense, it has been observed that some nano-sized additives added at low concentrations can generate significant improvements in the mechanical, thermal, optical, and physicochemical properties of the materials compared to the pure biopolymer, all this without compromising their biodegradability. The use of reinforcing materials with nanometric particle sizes favors a more homogeneous dispersion of these, increasing the specific surface of reinforcements such as nano cellulose and nanoclays; these materials also exhibit improved thermal, mechanical, and barrier properties when reinforcing concentrations used are lower (1–5% by volume) than when using micrometer size or larger reinforcements [9,10,11,12,13].

Recent research has focused on clay nano-particles, a type of reinforcement material that, when added to TPS, improves the mechanical performance of the polymer, resulting in materials with greater tension resistance toughness [14,15,16,17,18]. As nanoclays can be found in the form of overlapping layers, this arrangement makes it difficult for water molecules to diffuse through the material, causing better resistance to moisture and other gases [5,18,19]. Montmorillonite (MMT) is the most widely used natural clay, it is a layered silicate that is characterized by having a moderate negative surface charge. MMT’s typical crystalline chemical structure consists of two tetrahedral layers of silica interspersed with octahedral layers of magnesium or aluminum oxide’s shared edge, forming a two-dimensional mesh with a thickness of 1 nm. MMT has received a great deal of attention due to its complex layer structure, which provides it with high absorption capacity, good chemical stability, and the ability to of exchanging ions [20,21].

It has been reported that the final behavior of the nanocomposite material is highly dependent on the type of MMT-polymer interaction, i.e., if it is in exfoliated or intercalated form. Intercalation occurs when polymer chains are embedded between clay layers, resulting in a multilayer polymer-clay sheet structure. The silicate layers of the nanoclay have completely separated and are individually dispersed throughout the polymer matrix in the exfoliated form [22], which results in a greater surface area, promoting higher interaction between the MMT and the structural polymer matrix [23]. Conventional plastic processing techniques such as thermoforming, casting, or melt mixing do not achieve complete exfoliation of the nanoclay, according to some studies, so MMT interaction with the polymeric matrix only occurs in an intercalated form [24,25,26].

According to the foregoing, new biodegradable polymeric materials with improved mechanical and barrier properties are required. Nanoclays are seen as an excellent option for reinforcing these materials; however, an ideal process that promotes better dispersion of these nanomaterials must be developed. This new knowledge could help in the development of new active packaging with mechanical and gas barrier properties that rival synthetic plastics. In this study, biodegradable films based on thermoplastic starch/polyvinyl alcohol (PVA) reinforced with montmorillonite nanoclay (MMT) at different proportions were developed, processed by solvent casting, with and without the support of ultrasound, to evaluate the effect of this on the physical-mechanical properties of materials as a possible alternative as environmentally friendly food packaging.

## 2. Results

### 2.1. Surface Morphology by Light Optical Microscopy

**Figure 1:**
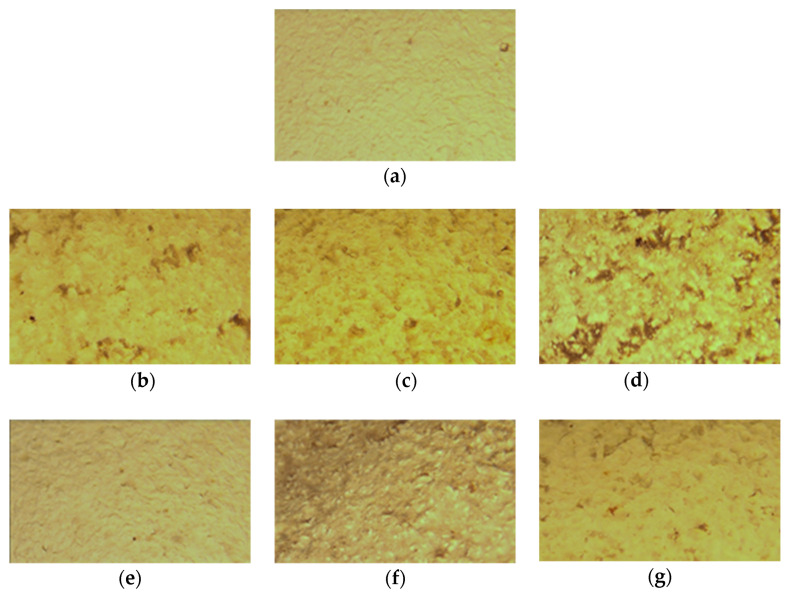
Optical microscopy images for (**a**) achira starch-PVA (SP) films with different concentrations of MMT (0.5, 1.0 and 1.5%), treated with (-s) and without sonication. 10× magnification. (**a**) SP, (**b**) SP0.5MMT, (**c**) SP1.0MMT, (**d**) SP1.5MMT, (**e**) SP0.5MMT-s, (**f**) SP1.0MMT-s, and (**g**) SP1.5MMT-s.

A conventional light optical microscope was used to observe the surface morphology of the achira starch/PVA (SP) composite films. According to the micrographs in Figure 1b,c, the film samples in each of the MMT formulations had a surface that was slightly even in appearance, but very different from the sample without nanoclay (Figure 1a). However, not all films had the same appearance; Figure 1d formulation had a less homogeneous surface behavior. In films containing 1.5 MMT, there were variations in the depth of the surface area, with a slightly rough appearance (Figure 1d).

Figure 1a–g show how the surface morphology of the resulting films changes when the filmogenic solutions are treated with ultrasound. A more homogeneous surface of the material is observed, probably due to a better ordering of the polymeric matrix with the reinforcing materials, which prevents the formation of structures such as those observed in samples without sonicating. This behavior can be attributed to an efficient dispersion of the MMT-Na nanoclay in the SP polymer matrix, which is a significant indicator of the effectiveness of the treatment and could be seen as an improvement in the functional properties of the nanocomposite films.

### 2.2. Surface Morphology by Scanning Electron Microscopy (SEM)

**Figure 2:**
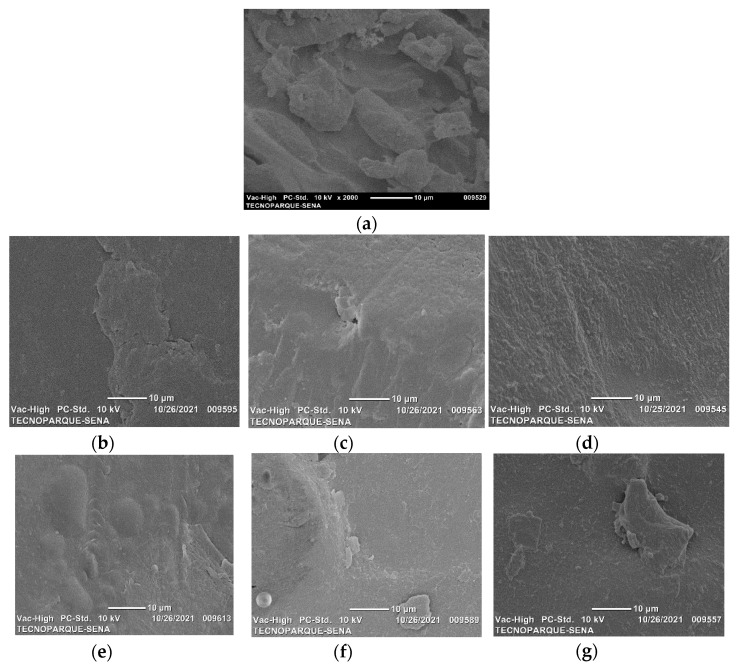
SEM micrographs for achira starch-PVA biodegradable films with MMT nanoclay (0.5, 1.0 and 1.5%), treated with (-s) and without sonication. (**a**) SP, (**b**) SP0.5MMT, (**c**) SP1.0MMT, (**d**) SP1.5MMT, (**e**) SP0.5MMT-s, (**f**) SP1.0MMT-s, and (**g**) SP1.5MMT-s. 2000× magnification.

**Figure 3:**
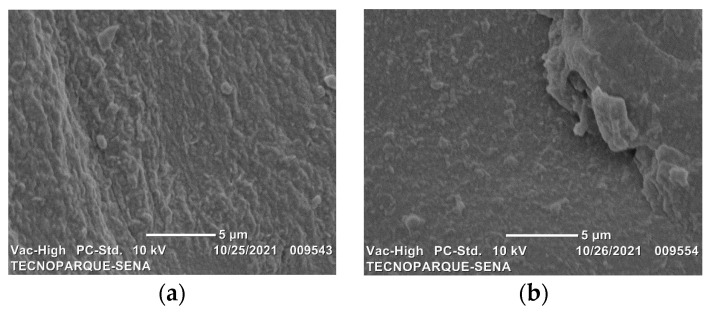
SEM micrographs for biodegradable films with MMT nanoclay at 5000× magnification. (**a**) SP1.5MMT and (**b**) SP1.5MMT-s.

Figure 2 presents the micrographs obtained on the fracture surface, these allow knowing the type of morphology both for the polymeric matrix and for the bionanocomposites. In the case of the polymer matrix without nanoclays (Figure 2a), the images indicate that the film is uniform, without phase separation. No droplets, pores or particles were observed. Structural integrity is due to the interaction between the hydroxyl groups of the biopolymers, which promotes the formation of hydrogen bonds. Deformation of a continuous phase typical of a ductile fracture (SP film) was observed when the nanoclays were added [27,28]. For the bionanocomposites with MMT (Figure 2b–d), the surface exhibits roughness and the presence of some aggregates as undispersed particles. This indicates that the fracture occurs across the interface between the load and the polymer matrix. When the amount of nanoclays in a mixture increases, the van der Waals forces favor the nanometric particles sticking together [29]. Scattered layers in sonicated samples (Figure 2e–g) could delay crack initiation due to a strong interaction with the matrix. Figure 3 shows a comparison between films with a higher content of nanoclay that show the effectiveness of the sonication process. SP1.5MMT-s film (Figure 3b) shows a surface with continuous film formations indicating excellent interaction between the clay layers and the polymer matrix. The cavitation phenomenon promotes the exfoliation of the nanosheets in sonicated samples, reducing the size of the aggregates and promoting a better dispersion within the polymer matrix.

### 2.3. Opacity

A material with good UV absorbing capacity can be an option for product packaging as opacity can help extend the shelf life of products. The optical properties related to translucency based on the integral over the maximum absorption peak values located for each mixture are shown in Table 1. The photos of each film (with a diameter of 5 cm) are displayed alongside the opacity values. The concentration of nanoclays has a direct relationship with these values. The behavior obtained is attributed to the fact that a higher load of nanoclays slightly increases the opacity of the materials. The SP1.5MMT sample presented the highest opacity, 230% more opaque than the SP film; while that same formulation when it is sonicated, the opacity increases 137%. According to the findings of various authors, opacity should not be significantly affected when the matrix and nanoclay are compatible. When clay plates are well dispersed in a polymeric matrix and have a size of less than 1 nm, they can allow light to pass freely without affecting the materials’ opacity [30].

### 2.4. FTIR-ATR Analysis

**Figure 4:**
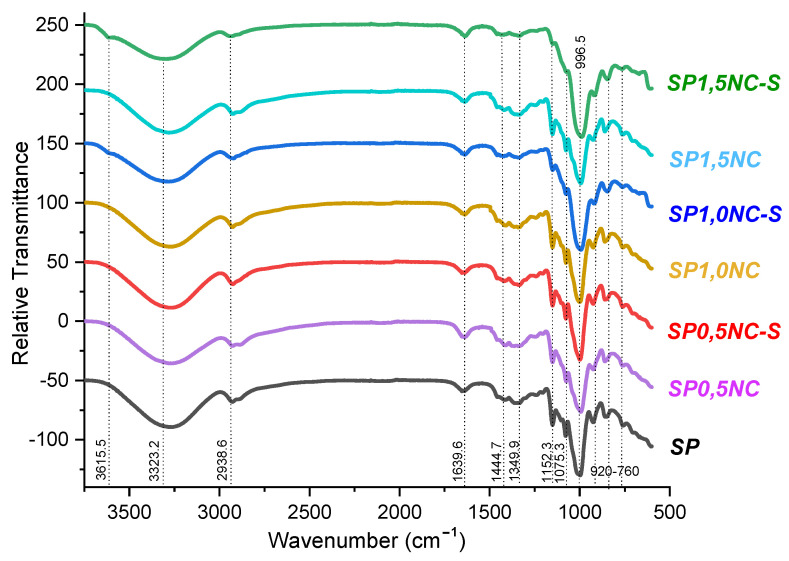
FT-IR spectra of bionanocomposite SP films at different concentrations of MMT clay and ultrasound treatment.

**Figure 5:**
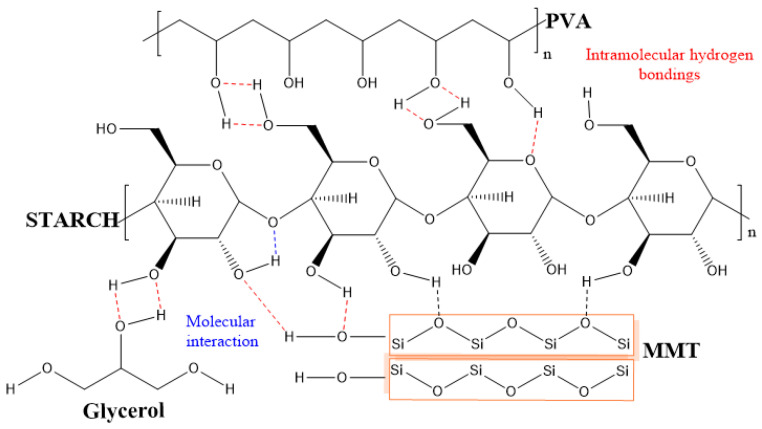
Schematic illustration of proposed molecular interactions between polymers (starch and PVA), plasticizer (glycerol), and MMT nanoclay.

To study the effects of the addition of nanoclays on the starch/PVA (SP) matrix structure due to possible interactions between starch, PVA, glycerol, and bentonite clay the FT-IR analysis was performed (Figure 4). The first band located at 627 cm^−1^ is associated with vibrations of the glucose rings’ skeletal model; similar findings have been reported by other authors [31,32]. Bands located from 920 cm^−1^ to 1153 cm^−1^ are associated with vibrations of C-C and C-O-H stretching and the bending mode of C-H bonds typical of vibrations of fingerprint of saccharides [31,33]. For example, the bands at 1075 and 1153 cm^−1^ are characteristic vibrations of C-O-H. However, both absorption bands are also associated with C-O-H of PVA. Thus, the decrease of these bands in films of SP1.0MMT-s and SP1.5MMT-s can be related to the OH vibrations being limited due to the strong interaction of the OH group of starch, PVA, and glycerol with the oxygen and OH of MMT nanoclay through a hydrogen bond. This result can be interpreted as a consequence of the high concentration of MMT nanoclay that reach to be dispersed along with the polymeric matrix, while in films of SP1.0MMT and SP1.5MMT was not possible to observe a reduction of the bands which means a bad dispersion of MMT nanoclay, also, the exfoliation of MMT helps to expose more easily OH and oxygen [34,35]. Added to this, there are other bands at 1444, 1639, 2938, 3323, and 3450 cm^−1^ that can be associated with the OH vibrations present in starch, PVA, and glycerol. As well, the band located at 2938 cm^−1^ could be attributed to hydrogen bonding interactions between the hydroxyl groups of both biopolymers. On the other hand, MMT nanoclays can be identified by stretching of Si-O-Si associated with bands of 631 cm^−1^ and 1075 cm^−1^ attributed to the bending of this group. These bands are related to the tetrahedral crystalline structure of the clay layers [36]. Similarly, the vibration of the terminal silanol groups (Si-OH) in MMT nanoclays is observed at 3615 cm^−1^ in films of SP1.0MMT-s and SP1.5MMT-s, meanwhile, in films with the same concentration of MMT nanoclays without sonication this band is not observed, this result is due to the sonication promotes the exfoliation of MMT and the Si-OH are exposed [34,37,38]. Figure 5 shows the possible molecular interactions between the biopolymers starch, PVA, and glycerol as a plasticizer, and MMT nanoclay.

### 2.5. Thermal Analysis

**Figure 6:**
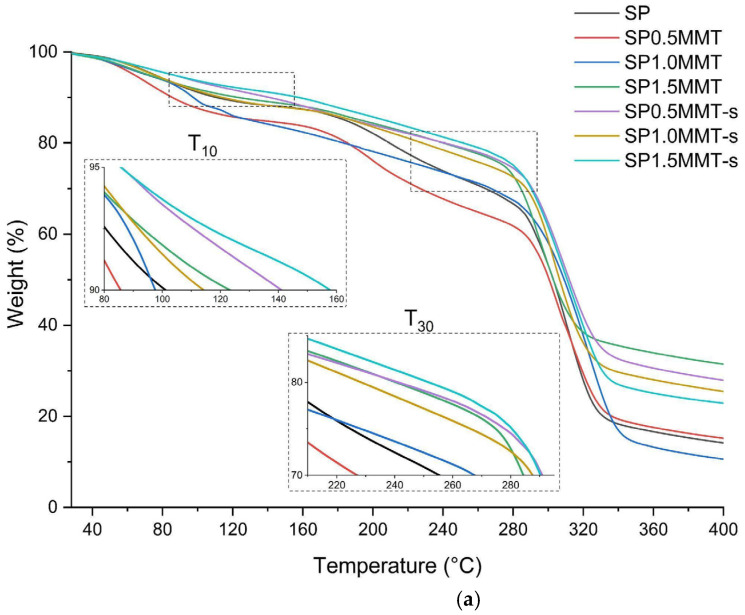

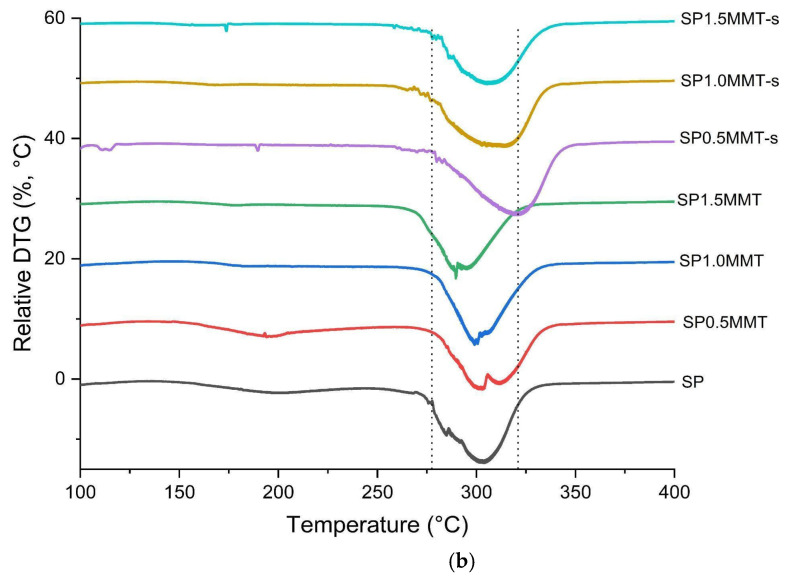
Thermogravimetric Analysis (TGA) (**a**) and Derivative Thermogravimetry (DTG) (**b**) curves of bionanocomposite films at different concentrations of clay and ultrasound treatment.

For the polymeric bionanocompounds, Figure 6 shows the curves obtained from TGA analysis and their respective derivatives. Three thermal events can be observed. The first occurs in the range of 80 °C to 200 °C and refers to the evaporation of the water contained in the starch. The hydrogen bonds formed by the hydroxyl groups (-OH) of glucose along its chain interact with the respective OH of water, allowing the starch to absorb a lot of water. The second stage corresponds to the evaporation of glycerol at temperatures between 200 °C and 280 °C. Finally, in the third stage, the degradation of starch is observed, in this step the elimination of polyhydroxy groups, oxidative degradation of products from the previous step, and polymer fragmentation [7,39]. Figure 6a shows two close-ups of the ranges between 80 °C to 160 °C and 200 °C to 300 °C; this allows determining the temperatures corresponding to a weight loss of 10% (T_10_) and temperatures prior to maximum degradation (with 30% weight loss, T_30_). When comparing ultrasound-assisted bionanocomposites to control films (SP), it is clear that the T_10_ was increased, and this trend was maintained for the other degradation temperatures. Similarly, the presence of clays in 1 and 1.5% without sonication treatment improves thermal stability. The effect with ultrasound is revealed when comparing SP0.5MMT with respect to SP0.5MMT-s, which increases thermal stability by ~100 °C indicated in the T_30_. Figure 6b shows a bifurcation in the maximum degradation band (~300 °C) that relates the elimination of water of crystallization contained in the clay and the degradation of the polymeric matrix [40]. The crystallization water molecules were evidenced in the non-sonicated bionanocompounds, these samples are more prone to form agglomerates since the nanometric size of the clay has a high internal surface. The sheets open due to cavitation in the ultrasound-assisted mixing of the filmogenic solution, facilitating the dispersion and exfoliation process of the clay sheets, limiting the formation of agglomerates [41]. This allows an increase in the maximum degradation temperature (T_d_) of around 10 °C for bionanocomposites with ultrasonic treatment.

Table 2 summarizes the results of the thermal analysis. In general, the nanocomposites without sonication showed a decomposition temperature close to that reported for the SP mixture with variations of ~5 °C. On the other hand, the sonicated bionanocomposites showed increases of 10 °C to 30 °C, for SP0.5MMT-s and SP1.0MMT-s, respectively. The thermal resistance that the incorporation of nanoclays promotes in polymeric materials has been widely reported [42]. The quality of the clay sheets’ dispersion process on the matrix, on the other hand, must be guaranteed. This will increase the surface area of the particles, allowing for more interaction. The infrared results are directly related to showing stronger interactions of SP with montmorillonite, which explains why starch is reorganized with fewer exposed hydroxyl groups, reducing susceptibility to heat degradation [20].

**Figure 7:**
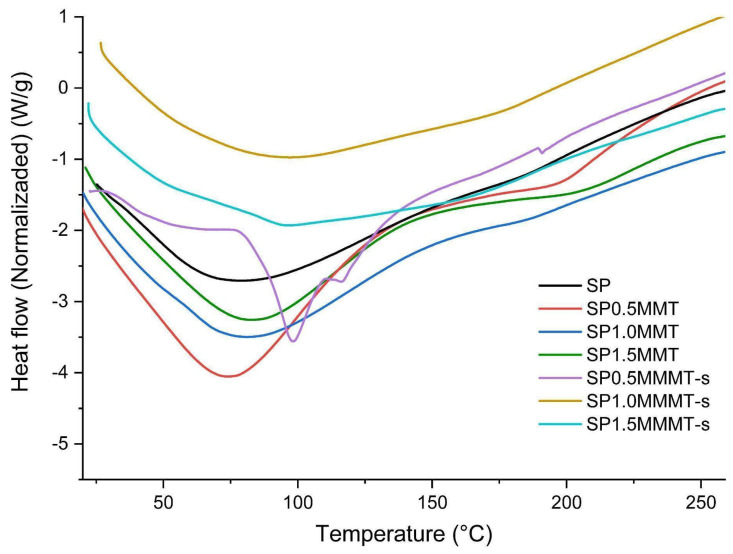
DSC thermograms of bionanocomposite films at different concentrations of clay and ultrasound treatment.

Figure 7 presents the DSC curves of the polymeric bionanocomposite films, where the first order and second-order transition temperatures are observed, related to the melting of each polymer (T_m_), gelatinization of the starch (T_gel_), and the glass transition of the PVA (T_g_), respectively. Second-order transitions appear as a large band between 35 °C and 98 °C, this broadening is due to the superposition of the T_g_ and T_gel_ indicated in Table 2.

T_gel_ of starch and non-sonicated nanocomposites is observed with greater definition between 74 °C and 82 °C, where probably the gelatinization of some granules was not completed. The band appears less intense, although the ultrasound-assisted samples increased by more than 10 °C during this transition. This indication shows that the ultrasound radiation on the solution promotes gelatinization. The melting temperature of the SP0.5MMT-s film was 116 °C, indicating that this material has a higher degree of molecular ordering than the other blends. The displacement of the peak indicates a strong interaction with the clay with the polymers, especially with the starch, generating a thermal insulating effect. Giannakas et al. obtained similar results using chitosan/PVA systems [43]. A synergistic effect was observed between the clay content and the sonication on the variables T_gel_ and T_g_ of PVA. Likewise, a melting of the PVA was evidenced at 180 °C, however, this transition did not present a clear trend.

### 2.6. Contact Angle and Water Absorption

**Figure 8:**
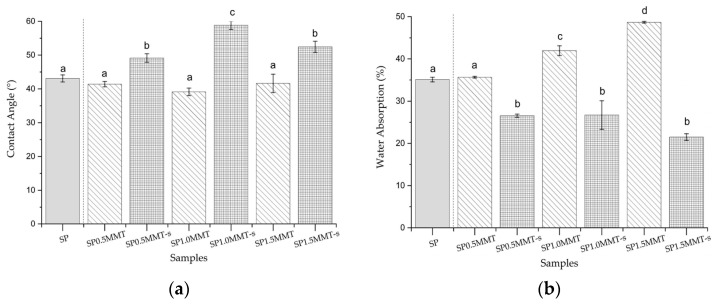
Effect of MMT nanoclay concentration and ultrasound treatment on (**a**) contact angle and (**b**) water absorption of starch/PVA (SP) composite films. SP control sample without nanoclay. Different letters (a, b, c, d) in the same column denote significant differences for the set of means in each treatment (with and without ultrasound) (*p* < 0.05).

The wettability and absorbent properties of films were evaluated and investigated at different contact angles and levels of water absorption. Figure 8 depicts the results of these tests. The incorporation of MMT nanoclay caused changes in both parameters, which could be seen. However, sonication of MMT nanoclay is crucial to understanding how the properties can be improved; films with sonicated MMT nanoclay had better wettability and absorbency. The highest values of contact angle concerning SP were presented by SP1.0MMT-s with an increase of almost 50%, thus reducing the hydrophilicity of the material. This can be explained by the possible ion-dipole interactions between the nanoclay with the OH groups of starch, water, and glycerol. These ion-dipole interactions cause a lower availability of hydroxyl groups, as well as a brief interaction (<30 s) with the SP films [44] observable as a higher contact angle. The balance between the cation-sheet electrostatic attraction and the cation’s hydration energy is crucial in this process. When sodium is the interlaminar cation, smectics have a high swelling capacity, and complete dissociation of individual smectite crystals can occur, resulting in a high degree of dispersion and maximum development of colloidal properties [45]. However, for the silicate to be uniformly distributed on the polymer matrix, the influence of ultrasound is required. The significant increase in contact angle observed with SP0.5MMT can be attributed to the formation of an aggregate on the surface, as evidenced by the obtained micrographs. The effect of increased water absorption in nanoclay films without sonication could be attributed to the interlaminar space of the nanoclay, this increase in spatial separation was observed as higher swelling (Figure 8b).

### 2.7. Water Vapor Permeability (WVP)

The nanostructure of the SP films reinforced with the MMT nanoclay creates a more tortuous path for the permeant gas to pass through, according to the evaluation of the materials to determine their water vapor barrier capacity. The water vapor molecules diffuse through the film, taking the path of least resistance. The most effective way to spread is the longer and more complicated channels between the layers of clay. Therefore, water vapor molecules travel perpendicular to diffusion by the most prolonged and most complex path. This process significantly reduces the gas diffuse rate, reflected in lower transmission (WVTR) and water vapor permeability (WVP) values (Table 3). However, although the WVTR was lower in the films with clay compared to those lacking this nanofiller (SP), the WVTR reduction effect was more noticeable in the films with the lowest clay content (SP0.5MMT-s). It was observed that adding more than 0.5% MMT to the polymeric achira starch/PVA (SP) formulations causes a significant increase in the WVTR, which is reflected in a decrease in the materials’ barrier capacity.

Even though films with a different proportion of nanoclay adsorbed similar amounts of water (Figure 8b), this behavior does not seem to influence the gas barrier behavior of the material in the same way. Even though the film formulated with 1.5MMT had lower water adsorption, its WVTR and WVP were significantly higher than the material were much higher than that of the materials with the lowest clay content (0.5). This suggests that the nano-reinforcements in each of the formulated films interact differently in the structural matrix.

This effect of improvement in the barrier capacity of the materials at low concentrations of nanoclay may be due to better dispersion and structural accommodation of the clay sheets in the polymeric matrix. Whereas when the concentration of MMT is higher, it becomes an excess, acting as an intermolecular free space increasing agent, making it difficult for other sheets in the matrix of the composite polymer is not longer possible, facilitating the passage of gases increasing their permeation.

### 2.8. Mechanical Properties

Table 4 presents the results of the tensile tests that were done to the sonicated samples and the control sample, for tensile strength (TS), percentage of elongation at break (ε), and Young’s modulus (YM). As has been reported by other authors [4,5,14,19,29], the addition of nanoclay increased the TS and YM for all nanoclay concentrations compared to the values obtained by the SP sample, where the greatest increase is presented by SP1.0MMT-s (24% tensile strength and 50% Young’s modulus). The clays’ nanometric size and high surface area facilitate the formation of an effective interface for transferring tensile stresses. Furthermore, the presence of higher-rigidity nanoclay can limit the polymeric matrix’s macromolecular mobility, increasing the nanocomposite films’ elastic modulus [46] favoring a ductile tearing of the material, where the fracture occurs across the interface between the polymer and the reinforcement (Figure 2e–g). Therefore, when the crack meets a reinforcing particle, which is weakly adhered, the energy of the crack is dissipated and prevents its propagation, generating a plastically deformed surface due to tensile stresses, on the other hand, the exfoliation of the nanoclay and its good dispersion in the matrix, can delay the onset of the crack due to the strong interaction of its compounds. These same results were found by Tin et al. [47] by showing that both mechanical properties were increased by the presence of MMT in blends of PVA/starch with 0 to 25% of MMT.

Higher nanoclay contents, on the other hand, as demonstrated by SEM in Figure 2g favor the formation of aggregates, resulting in a deterioration of these properties in the SP1.5MMT-s sample, because the large size of the aggregates they become defects that are stress concentrators, leading to early fracture of the material, because the large size of the aggregates they become defects that are stress concentrators, leading to early fracture of the material.

Since the interaction of starch with the crystallization water molecules contained in the nanoclay generates a plasticizing effect that increases its elongation capacity, the SP0.5MMT-s sample has a 35% increase in elongation capacity when compared to the SP sample. A higher addition of nanoclays did not significantly modify this property in the films SP1,0MMT-s and SP1,5MMT-s. This higher addition of MMT favors the formation of new nucleation points that contribute to the growth of the polymer crystals, causing more brittle films. This is consistent with the result obtained by Müller et al., who developed starch nanocomposites with two types of nanoclay and two different concentrations, where, the control presents a maximum elongation of 63 ± 12%, while when including the nanoclays the values are between 74 and 76%, evidencing in all cases the low incidence on ε [4].

## 3. Materials and Methods

### 3.1. Materials

The following biopolymers were used: Achira starch by Surtialmidon (Huila, Colombia) with a density of 1.59 g/mL; Polyvinyl alcohol (PVA) from Fluka Analytica (Prague, Czech Republic) (Mw = 47,000 g/mol), with a polymerization degree of 1000 and 98% of hydrolysis. Montmorillonite nanoclay (MMT), hydrophilic bentonite density of 600 to 1100 kg/m^3^, average particle size ≤ 25 µm by Sigma–Aldrich (St. Louis, MO, USA) and, Glycerol with a density of 1.26 g/mL (purity: 99.68%).

### 3.2. Film Preparation

Gómez-Aldapa et al. (2020) proposed a formulation and methodology for the production of biodegradable films, which was used in this study [7]. The PVA solution was prepared by dissolving the PVA in distilled water at a concentration of 4% (*w*/*v*) and heated to 70 °C and kept at that temperature under stirring for 2 h. The corresponding glycerol (25% *w*/*w*, total polymers) was added in distilled water and stirred for 5 min for the nanocomposite films of starch-PVA-nanoclay. The achira starch was added and stirred until a temperature of 50 °C was reached after the plasticizer had dissolved. The corresponding PVA solution was incorporated after the temperature was reached, and the nanoclay (0.5, 1.0, and 1.5 % *w*/*v*) dispersed in water was added (see Table 5). For 5 min, the solution was kept under constant stirring and the temperature was raised to 85 °C to gelatinize the starch. The filmogenic solution was treated with an ultrasonic water bath (Elmasonic Easy 120H, Elma Schmidbauer GmbH, Singen, Germany, at a fixed frequency of 37 kHz and power of 200 W) for 5 min at 85 °C to favor the dispersion of the nanoparticles. The filmogenic solution obtained was poured into a Petri dish covered by a Teflon sheet and dried for 5 h in a humidity chamber at 65 °C and 50% relative humidity. The thickness of the films was 180 ± 20 μm. The films were kept at 25 °C and 50% relative humidity prior to analysis.

### 3.3. Characterization

#### 3.3.1. Optical Microscopy

Optical microscopy with an Olympus BX60 light microscope (Tokyo, Japan) at 10× magnification was used to determine the surface morphology of films at low magnification. The films were dried at room temperature and immobilized on a glass slide.

#### 3.3.2. Scanning Electron Microscopy

The tensile fracture surfaces (surface perpendicular to the tensile loading direction) of the films was analyzed at higher magnification using a JEOL, JCM 50,000 (Tokyo, Japan) scanning electron microscope (SEM). A voltage of 10 kV was applied. The samples were covered with a gold layer. The surface films were analyzed at 2000× and 5000× magnification.

#### 3.3.3. Opacity

Mali and Grossmann’s proposed methodology was used to determine the opacity of the films [48]. Composite films were cut at 1 × 3 cm and placed inside a Shimadzu UV-VIS 2600 spectrophotometer cell (Columbia, MD, USA), samples were measured between 400 nm–800 nm. The area under the curve was defined as absorbance units × nanometers (AU nm), and the opacity was defined as the area under the curve. Duplicate measurements were taken.

#### 3.3.4. Fourier Transformed Infrared Spectroscopy (FT-IR)

FT-IR analysis of films was carried out by ATR mode using a spectrophotometer Spectrum 3 by PerkinElmer (Waltham, MA, USA). Dry films were analyzed by 16 scans, a wavenumber range between 600 cm^−1^ and 4000 cm^−1^, and a 4 cm^−1^ resolution.

#### 3.3.5. Thermal Properties

Thermal stability of films was determined by thermogravimetric analysis (TGA) using a TGA/DSC 2 STAR System instrument, Mettler Toledo, Columbus, OH, USA. The sample was heated from 25 °C to 600 °C at a heating rate of 20 °C/min under a nitrogen purge at a flow rate of 60 mL/min. The weight loss was shown as a function of temperature.

Differential Scanning Calorimetry (DSC) was used with TA Q-2000 equipment (TA Instruments, New Castle, DE, USA), to identify the thermal transitions of films at a heating rate of 10 °C/min in a temperature range from 25 °C to 200 °C with a nitrogen purge.

#### 3.3.6. Contact Angle

At room temperature, the contact angle was measured with a goniometer Ramé-Hart Model 250 (Netcong, NJ, USA). A drop of distilled water (20 µL) was dropped onto the surface of the sample before measuring the contact angle of the water as a function of time. The measurement was performed three times. ANOVA with a significance level of 0.05 was used for the statistical analysis.

#### 3.3.7. Water Absorption

To study the water absorption behavior of the films, they were cut to a size of 2 × 2 cm and dried for 4 h at 50 °C, later; films were weighed and soaked in distilled water for 6 h at room temperature, subsequently, the weight of the drained films was taken. The water absorption was calculated with the Equation (1):

(1) $$\%W=\frac{W_{t}-W_{0}}{W_{0}}\times 100$$

where *W_t_* is the weight of films after the exposed time interval and *W_0_* is the initial dry weight of the sample.

#### 3.3.8. Water Vapor Transmission Rate (WVTR) and Water Vapor Permeability (WVP)

The water vapor transmission rate (WVTR) was performed at a Permatran 3/33 apparatus (Mocon Inc., Minneapolis, MN, USA). The test conditions were: film sample area of 5 cm^2^, 25 °C, 50% RH, and the partial pressure of oxygen was 760 mmHg. To determine the WVP, the WVTR was normalized to the film thickness (180 μm). The mean value was calculated from three replicates for each film formulation. Only films made from filmogenic solutions that had been sonicated were used for this analysis.

#### 3.3.9. Tensile Test

A tensile test was performed on the mechanical properties using a universal machine INSTRON model EMIC 23–50 (São José dos Pinhais, PR, Brazil) equipped with a 50 kN load. The measurements were done on 6 specimens for each sample with dimensions of 10 × 2.5 cm and a thickness variable, using a constant rate of 1 cm/min until rupture. The samples were conditioned previously at 23 °C for 2 days. Only materials obtained from sonicated film-forming solutions, such as the WVP test, were used for this characterization.

### 3.4. Statistical Analysis

Analysis of variance (ANOVA) was performed by Tukey’s test (0.05 level of significance) to compare mean differences of the nanocomposite films formulations. All statistical analyzes were performed with IBM SPSS Statistics for Windows version 25 (New York, NY, USA).

## 4. Conclusions

In this study, achira starch/PVA (SP) films reinforced with MMT nanoclay were developed in 0.5–1.5% concentrations, with and without ultrasound sonication, obtained by the solvent casting method. Due to the strong interactions between the biopolymeric blend matrix and clay nanoparticles, platelets are more effective when processed in the presence of ultrasound, as verified by FT-IR and SEM analyses. The use of an ultrasound treatment generated materials with a better, more homogeneous surface, as observed with the SEM, as well as a reduction in the water susceptibility of the films by contact angle and water absorption. Due to the insulating effect of the nanoclays present, the thermal properties of the nanocomposite biomaterials revealed an improvement in thermal stability compared to the biopolymer mixture (SP). Strong intermolecular interactions were observed as thermal transitions at higher temperatures in the ultrasonic-treated samples. Furthermore, the SP0.5MMT-s film showed a complete plasticization with respect to the non-sonicated homologue (SP0.5MMT), also against the SP control. The incorporation of the MMT nanoclay had a reinforcing effect, improving the SP film’s tensile stress mechanical properties and Young’s modulus. Likewise, it is possible to obtain composite materials reinforced with MMT with different water vapor barrier behaviors, which can be achieved by increasing the concentration of the nanoclay. At low concentrations of MMT, a less gas-permeable material is obtained, but when the percentage is 1.0 or higher, the material becomes more permeable.

These results demonstrate that nanoclays are promising nanoreinforcers for improving the functional properties of achira starch/PVA (SP) blend packaging materials and forming a new sustainable material for potential application in the packaging sector. Combining these new approaches provides the necessary innovation capable of potentiating the migration towards more sustainable materials with shorter degradation times and the required performance characteristics for their application in different industrial sectors.

## Figures and Tables

**Table 1:** Opacity values and real images of the appearance of the SP films to the human eye of polymeric bionanocomposite films.

Sample	Opacity (AU × nm)	Pictures	Sample	Opacity (AU × nm)	Pictures
SP	12.51 ± 0.02 ^a^ (at 400 nm) *	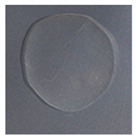			
SP0.5MMT	12.96 ± 0.09 ^a^ (at 510 nm) *	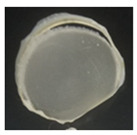	SP0.5MMT-s	12.55 ± 0.09 ^a^ (at 510 nm) *	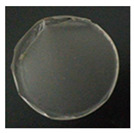
SP1.0MMT	23.28 ± 0.12 ^b^ (at 590 nm) *	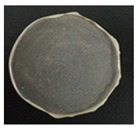	SP1.0MMT-s	15.26 ± 0.08 ^c^ (at 590 nm) *	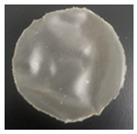
SP1.5MMT	28.87 ± 0.17 ^b^ (at 590 nm) *	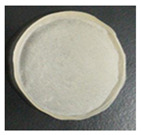	SP1.5MMT-s	17.12 ± 0.11 ^c^ (at 590 nm) *	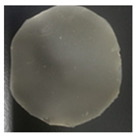

* Maximum absorption peak measured. SP control sample without nanoclay. Different letters (^a^, ^b^, ^c^) in the same column denote significant differences for the set of means in each treatment (with and without ultrasound) (*p* < 0.05).

**Table 2:** Weight losses of 10% (T**_10_**), initial degradation (T**_i_**), maximum degradation (T**_d_**), final degradation (T**_f_**), glass transition (T**_g_**) and melting (T**_m_**) temperatures, results based on the obtained TGA and DSC thermograms for the bionanocomposites.

Sample	T_10_	T_i_	T_d_	T_f_	T_gel_	T_m (SP)_	T_g (PVA)_
	(°C)						
SP	105.0	264.4	297.3	329.5	77.8	--	--
SP0.5MMT	85.3	271.7	300.3	336.1	74.5	--	196.6
SP1.0MMT	97.2	268.8	295.9	335.5	81.1	--	184.8
SP1.5MMT	122.5	268.1	292.3	334.7	82.6	--	203.0
SP0.5MMT-s	140.6	269.5	306.2	342.8	98.1	116.4	191.3
SP1.0MMT-s	113.0	271.2	327.7	353.7	96.0	--	182.8
SP1.5MMT-s	156.8	279.0	312.7	346.4	96.1	--	170.9

**Table 3:** Water vapor transmission rate (WVTR) and water vapor permeability (WVP) of starch-polyvinyl alcohol (SP) film reinforced with nanoclays (MMT), sonicated with ultrasound.

Film Sample	WVTR g·mm/(m^2^·day)	WVP g/(m^2^·day)
SP	1073.2170	4658.290
SP0.5MMT-s	331.3667	3122.959
SP1.0MMT-s	736.2055	3675.283
SP1.5MMT-s	884.4438	4836.468

**Table 4:** Mechanical properties of SP films reinforced with nanoclays.

Sample	TS (MPa)	ε (%)	YM (MPa)
SP	3.73 ± 0.30 ^a^	39.45 ± 10.9 ^a^	0.179 ± 0.01 ^a^
SP0.5MMT-s	3.87 ± 0.32 ^a^	61.39 ± 9.6 ^b^	0.138 ± 0.02 ^a^
SP1.0MMT-s	4.89 ± 0.37 ^b^	33.16 ± 7.3 ^a^	0.360 ± 0.05 ^b^
SP1.5MMT-s	4.75 ± 0.25 ^b^	34.54 ± 4.2 ^a^	0.328 ± 0.03 ^b^

TS: tensile strength, ε: elongation at break, YM: Young’s modulus. a–b Different letters (^a^, ^b^) in the same column indicate significant differences (*p* < 0.05). Mean of five replications ± standard deviation.

**Table 5:** Composition of the films based on achira starch, and polyvinyl alcohol, with MMT nanoclay.

Sample *	Starch (%)	PVA (%)	Nanoclay (%)	Mixing Method
SP (control)	60	40	0	Stir
SP0.5MMT	60	40	0.5	Stir
SP1.0MMT	60	40	1.0	Stir
SP1.5MMT	60	40	1.5	Stir
SP0.5MMT-s	60	40	0.5	Stir and sonication
SP1.0MMT-s	60	40	1.0	Stir and sonication
SP1.5MMT-s	60	40	1.5	Stir and sonication

* Abbreviation: thermoplastic achira starch = S, polyvinyl alcohol = P, montmorillonite nanoclay = MMT, and sonication = s.

## Data Availability

The data are available on request from the corresponding author.

## Sample Availability

Samples of the compounds are available from the authors.
